# Incidental Finding of a Fish Bone Perforation in the Pylorus Mimicking Acute Cholecystitis

**DOI:** 10.7759/cureus.54596

**Published:** 2024-02-21

**Authors:** Chai Wei Tong, Sam Alhayo, Francis Chu

**Affiliations:** 1 Department of Surgery, St. George Hospital, Sydney, AUS; 2 Faculty of Medicine and Health, University of New South Wales, Sydney, AUS; 3 Department of Upper Gastrointestinal Surgery, St. George Hospital, Sydney, AUS; 4 Department of Upper Gastrointestinal Surgery, University of New South Wales, St. George and Sutherland Clinical School, Sydney, AUS

**Keywords:** gastrointestinal perforation, sharp foreign body ingestion, endoscopic removal, upper gastrointestinal surgery, foreign body ingestion in adults

## Abstract

We present a case of a 43-year-old man with Crohn’s disease who presented with epigastric and right upper quadrant abdominal pain, initially suspected to be acute cholecystitis or a Crohn’s flare-up. CT revealed a curvilinear, hyperdense foreign body adjacent to the duodenum, concerning micro-perforation. Endoscopic examination confirmed findings of a 3 cm fish bone lodged in the pylorus. Endoscopic extraction was successful without significant mucosal damage, and the patient recovered well postoperatively. This case highlights the rarity of pyloric perforation secondary to fish bone ingestion and highlights the importance of considering this diagnosis in patients presenting with unexplained acute abdominal pain, as prompt recognition and intervention are essential for favorable outcomes.

## Introduction

Accidental ingestion of fish bones is a common incident in emergency departments; the vast majority spontaneously pass through the digestive tract without complications; however, a less than 1% subset can result in perforation. Perforation of the pylorus from fish bone is rare, and so far only two cases in Australia have been reported [1]. Here we present a case of pyloric perforation mimicking acute cholecystitis.

## Case presentation

**Figure 1:**
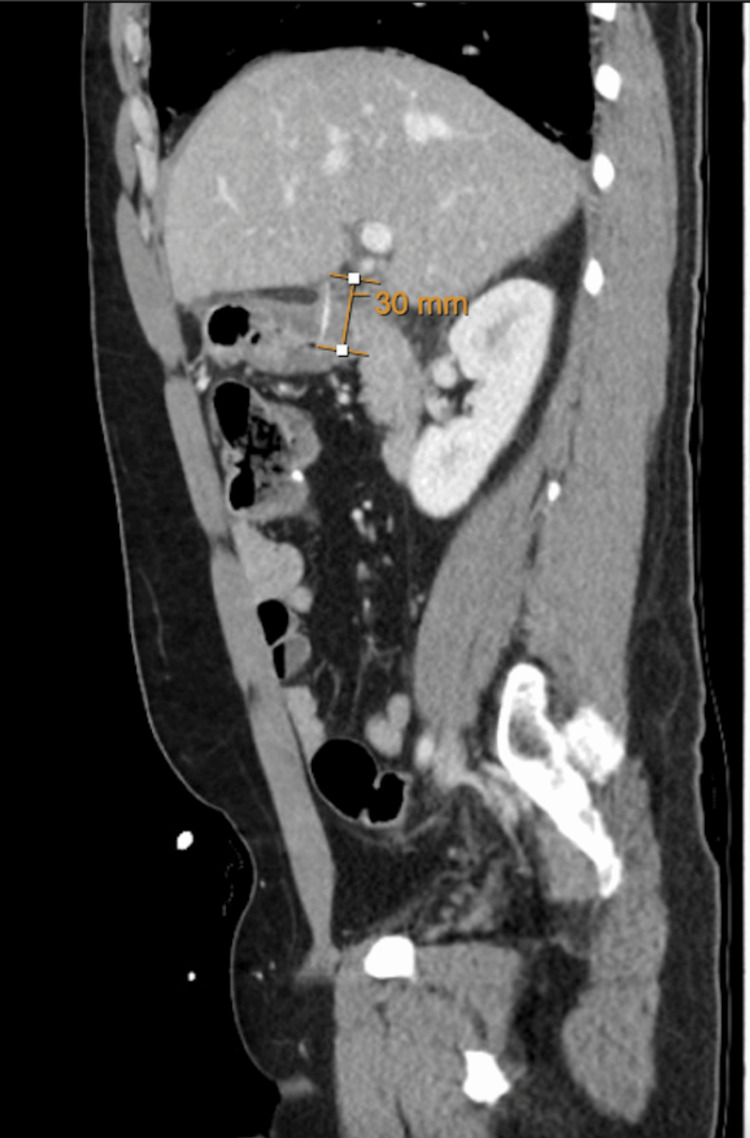
A 3 cm curvilinear hyperdense foreign body extending from the first part of the duodenum appears to perforate the superior wall of D1. The foreign body approximates the wall of the gallbladder, and the tip indents the undersurface of the adjacent segment 4B of the liver

**Figure 2:**
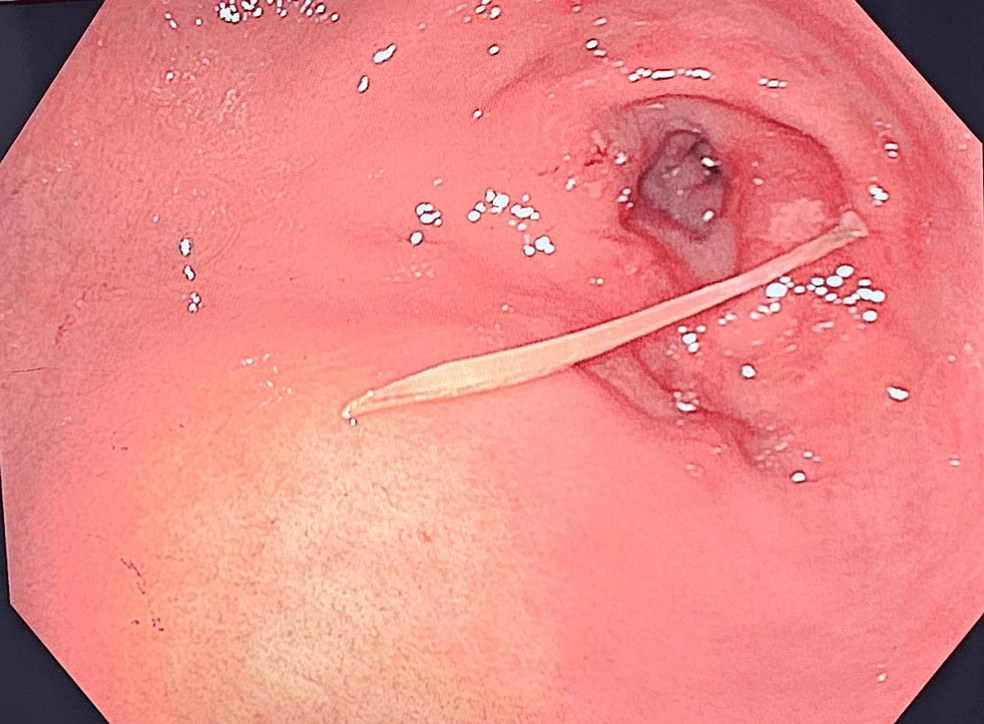
Endoscopic view of the surrounding mucosa following extraction of the fish bone

A 43-year-old man with a background of Crohn’s disease presented with a two-day history of epigastric and right upper quadrant abdominal pain, associated with fevers and loose stools. A physical examination showed epigastric tenderness with localized peritonitis. Laboratory examination showed a white cell count of 11.2x10^9/L and a raised C-reactive protein level of 180 mg/L, as well as a raised bilirubin level of 37 µmol/L. The initial list of differential diagnoses included acute cholecystitis or a flare-up of Crohn’s disease, but a CT identified a 3 cm curvilinear hyperdense foreign body around the first part of the duodenum, with moderate fat stranding in the right upper quadrant next to the gallbladder, suspicious for a micro-perforation (Figure 1). There was no evidence of pneumoperitoneum. On retrospective history-taking, the patient admitted to having consumed fish prior to the onset of symptoms. The patient underwent endoscopic examination under general anesthesia, and a 3 cm-long fish bone (Figure 2) was found lodged in the pylorus with near-complete penetration through the mucosa. The fish bone was successfully retrieved using a pair of endoscopic forceps. There was no significant mucosal defect or bleeding of the pylorus following the removal of the fish bone. The patient was kept in the hospital for further observation and antibiotics. He was able to tolerate a liquid diet without any abdominal pain and was successfully discharged home on postoperative day three to continue two weeks of a soft diet.

## Discussion

Accidental ingestion of fish bones is a prevalent occurrence encountered frequently in emergency departments. Fortunately, the majority of these incidents result in the spontaneous passage of fish bones through the digestive tract, which are excreted in the stool without causing significant complications. Studies have shown that more than 99% of ingested foreign bodies navigate the gastrointestinal system without any problems, emphasizing the generally benign nature of such cases [2]. However, a small subset, accounting for less than 1% of cases of ingested fish bones, can lead to perforation, abscess formation, mucosal ulceration, and peritonitis. It is noteworthy that the terminal ileum is the most frequently reported site of perforation, closely followed by the duodenal C-loop. The duodenum's retroperitoneal position, relative rigidity, and presence of deep transverse rugae and sharp angulations predispose it to entrapment of long and sharp-ended objects [3-5].

Due to the various potential sites of perforation, clinical diagnosis of foreign body perforation can be difficult as symptoms can mimic other more common presentations of acute abdominal pain; for example, symptoms of right upper quadrant pain are more common in cholecystitis, and right lower quadrant pain is usually suggestive of appendicitis [6-7]. CT is a highly effective imaging modality when the ingestion of a foreign body is suspected. CT is sensitive in locating and identifying non-radio-opaque foreign bodies and also plays a vital role in identifying potential complications such as perforation or abscess [8-9].

The European Society of Gastrointestinal Endoscopy recommends urgent endoscopic management for sharp foreign bodies in the upper gastrointestinal tract due to the risk of perforation and complications, as mentioned above [10]. Various studies have shown that endoscopic removal of sharp-pointed foreign bodies in the gastrointestinal tract is safe and effective. Surgical exploration of an embedded foreign body in the duodenum can be technically challenging, given the anatomy of the duodenum as well as in scenarios where the lumen is not perforated completely [11-12]. Compared to surgical removal, endoscopic foreign body removal is generally associated with fewer adverse events, less tissue trauma, and lower blood loss [12]. However, in patients with large perforations, peritonitis, or failure of endoscopic removal of a foreign body, surgical exploration with diagnostic laparoscopy or laparotomy should be considered [13].

## Conclusions

Acute intestinal perforation resulting from fish bone ingestion is uncommon but constitutes a medical emergency. The rarity of such cases often presents a diagnostic dilemma. This case report highlights the importance of recognizing foreign body perforation as a differential diagnosis in patients with unexplained acute abdominal pain, fevers, and a history of ingestion of foreign bodies. Endoscopic intervention by an experienced endoscopist is valuable in facilitating the extraction of foreign objects with minimal complications.
